# Loss to follow-up in a cohort of HIV-negative men who have sex with men: Project Horizonte

**DOI:** 10.1590/S1518-8787.2017051006681

**Published:** 2017-06-20

**Authors:** Ana Paula Silva, Marília Greco, Maria Arlene Fausto, Mariângela Carneiro

**Affiliations:** I Programa de Pós-Graduação em Ciências da Saúde: Infectologia e Medicina Tropical. Faculdade de Medicina. Universidade Federal de Minas Gerais. Belo Horizonte, MG, Brasil; II Projeto Horizonte. Universidade Federal de Minas Gerais. Belo Horizonte, MG, Brasil; III Departamento de Alimentos. Escola de Nutrição. Universidade Federal de Ouro Preto. Ouro Preto, MG, Brasil; IV Departamento de Parasitologia. Instituto de Ciências Biológicas. Universidade Federal de Minas Gerais. Belo Horizonte, MG, Brasil

**Keywords:** Men, HIV Long-Term Survivors, Homosexuality, Male, Bisexuality, Lost to Follow-Up, Cohort Studies

## Abstract

**OBJECTIVE:**

The objective of this study is to estimate the attrition rates and evaluate factors associated with loss to follow-up between 1994 and 2011 in an open cohort of HIV-negative men who have sex with men.

**METHODS:**

The Project Horizonte is an open cohort study that aimed to assess the incidence of HIV infection, evaluate the impact of educational interventions, and identify potential volunteers for HIV vaccine trials. The rates of losses to follow-up were estimated for three periods (1994–1999, 2000–2005, and 2006–2011). The variables analyzed were collected in a psychosocial questionnaire. Volunteers who dropped out were compared with the ones who remained in the study using a Cox regression model.

**RESULTS:**

A total of 1,197 volunteers were recruited. The median follow-up time in the study (n = 626) was 4.2 years. The median follow-up time for the volunteers who dropped out of the study (n = 571) was 1.46 years. The overall rate of loss to follow-up was 11.6/100 person-years. Attrition rates by period were: 12.60 (1994–1999), 11.80 (2000–2005), and 9.00 (2006–2011) per 100 person-years. Factors associated with losses to follow-up were: age group of 21–30 years old, monthly *per capita* income of more than six or less than one Brazilian minimum wage, having more than two dependents, report of bisexual practice, and inconsistent use of condoms for receptive anal sex.

**CONCLUSIONS:**

A slight decrease of the loss to follow-up was observed over time. Higher attrition rates happened in the first three years of follow-up. It is possible that the link of the volunteers were not yet well established. Those who reported inconsistent condom use in receptive anal sex were more likely to leave the study, suggesting an underestimation of the incidence of HIV infection in a cohort population. For greater effectiveness, retention strategies must be reassessed considering the connection between the characteristics of homosexual and bisexual behavior and the motivations to engage in health research.

## INTRODUCTION

It is estimated that 686,478 individuals were living with HIV/AIDS in Brazil in 2012, which represents a prevalence rate of 0.4% of the population. The epidemic in the country is concentrated in populations at higher risk of HIV exposure, including sex workers, injecting drug users, and men who have sex with men (MSM). The prevalence of infection among MSM is 11.1%, with higher prevalence among individuals aged 15–24 years^1^.

The Project Horizonte was an open cohort study of MSM, established in Belo Horizonte, State of Minas Gerais State, Brazil, in 1994. The objectives of the Project Horizonte were to establish and follow a HIV-negative MSM open cohort to (i) evaluate the feasibility of following such a cohort for an extended period of time, (ii) determine HIV incidence, (iii) evaluate counseling and educational practices for reducing risk, (iv) evaluate the possibility of conducting clinical trials of preventive HIV vaccines with cohort members, and (v) discuss ethical and technical aspects of clinical trials with preventive HIV vaccines. The Project Horizonte is the single running cohort of HIV-negative MSM in Brazil^2^.

One of the challenges of cohort studies is to minimize the attrition rate or loss to follow-up. Potential analytical bias can occur because of possible differences between the lost population and the individuals who are being followed with respect to the variables of outcome or exposure of interest^3^ ^-^ ^5^.

Few studies assessing factors associated with loss to follow-up among MSM are available in the literature. In most studies, the main factors associated with the loss reported sociodemographic characteristics and some behavior variables, such as smoking, drug use, and alcohol intake^3^ ^,^ ^6^ ^-^ ^8^. There is no systematic assessment of risky sexual practices and socio-behavioral factors associated with the attrition rates in this population.

In addition, it is important to know the determinants of losses to follow-up among participants in cohort studies because these losses can compromise the external validity, limiting the generalization of the results^7^ ^,^ ^9^ ^-^ ^10^. Therefore, this study aimed to estimate the attrition rates during follow-up and evaluate factors associated with loss to follow-up among MSM enrolled in the Project Horizonte in the period between 1994 and 2011.

## METHODS

The protocol of the Project Horizonte, concurrent cohort study of MSM, consists of two phases: screening process (recruiting and admission) and follow-up^2^ ^,^ ^11^. During recruiting, the aim was to identify men who have sex with men, HIV-negative, and over 18 years of age. In this phase, the psychosocial team interviewed the volunteer and described the study plan and objectives. Snowball sampling has been the main source of recruitment. The strategies of visibility used were pamphlets in lesbian, gay, bisexual, and transgender (LGBT) places; partnerships with non-governmental or governmental organizations; inserts in mainstream media, and team composed of Project Horizonte volunteers for disclosure to the LGBT community.

In the follow-up phase, the participants were evaluated semiannually to detect incidence of HIV infection, investigate risk factors for infection, and evaluate counseling and educational practices for reducing risk. The visits included a psychosocial interview using a semi-structured questionnaire with 95 questions that included sociodemographic variables, sexual practices, types of partnership, use of condoms, sexual violence, risk perception, use of alcohol or drugs, and knowledge and motivation for participating in future HIV vaccine trials. Technical and ethical aspects of HIV vaccine trials were discussed, and laboratory tests were prescribed (HIV serology, syphilis, hepatitis B and C). Serological results were provided by the psychosocial team with post-test counseling. These tests were followed by a clinical appointment. All exam data were recorded on a standardized clinical form. Preventive group interventions were implemented during the follow-up period, such as discussion forums, workshop on safe sex, and showing of films following the discussion. During these activities, issues on sexuality, sexual health, prevention, and HIV vaccines were discussed. According to the protocol of the Project Horizonte, the volunteers are followed for five years with semiannually visits.

Participants admitted to the Project Horizonte between 1994 and 2011 were selected for this analysis. Overall attrition rates were estimated at different periods of the follow-up. The factors associated with the loss to follow-up were evaluated by comparing the participants who dropped out the study at different times during follow-up with those who remained in the study.

The outcome of interest was loss to follow-up. Non-attendance at four consecutive follow-up visits (two-year follow-up) was considered loss to follow-up.

The variables analyzed were selected from the psychosocial questionnaire administered at the last visit for both those who dropped out the cohort and those who remained in the study. Data refer to the six months preceding the interview. The following variables were included:

- Sociodemographic variables: age, marital status, education, occupation, self-reported race, monthly income, and access to health services.
- Sexual behavior variables: type of partnership (steady or casual), sex of the partner, type of sexual practice (insertive or receptive anal sex), number of partners, condom use (consistent or inconsistent), transactional sex (sex in exchange for money or favors), sexual behavior (homosexual or bisexual).
- Contextual variables: contextual variables refer to the relationship of individuals and their sociocultural context and included places to look for sexual partners, use of alcohol or drugs, risk perception, willingness to participate in HIV vaccine trials.

Types of partnership were defined as follows: steady – when subjects reported emotional involvement and continuity of encounters (not necessarily based on partnership duration) – and casual – when subjects reported lack of emotional involvement or uncertainty of a recurring encounter, including strangers. Steady and casual partnerships were considered separately, considering that subjects could report either type of partnership, or both.

Condom use was classified as consistent (condoms are always used) and inconsistent (condoms are not always used). The inconsistent use of condoms was classified according to the type of sexual practice and the type of partnership. The consistent use of condoms was associated with the type of sexual practice.

Overall and annual attrition rates per 100 person-years (95%CI) were estimated (Table 1). To assess the calendar effect on the losses to follow-up, the attrition rates were calculated by grouping the individuals according to their year of recruitment (1994–1999; 2000–2005; 2006–2011) in the Project Horizonte (Figure). Individuals with only one follow-up visit and those who attended the follow-up were censored at the last visit, adding the expected average time between visits (0.25 years).

**Table 1:** Follow-up periods, losses to follow-up, and attrition rates per 100 person-years. Project Horizonte, 1994–2011.

Follow-up (years)	Person-time	Losses to follow-up (n)	Attrition rate (100 person-years)	95%CI
0 ┤1	1,016.85	233	22.91	20.15–26.05
1 ┤2	775.24	104	13.42	11.07–16.26
2 ┤3	609.35	74	12.14	9.67–15.25
3 ┤4	490.38	38	7.75	5.64–10.64
4 ┤5	405.50	30	7.40	5.18–10.58
5 ┤6	342.02	26	7.60	5.18–11.16
6 ┤7	276.38	17	6.15	3.82–9.89
7 ┤8	226.93	11	4.85	2.68–8.75
8 ┤9	185.94	16	8.60	5.27–14.04
9 ┤10	146.49	11	7.51	4.16–13.56
> 10	446.69	11	2.46	1.36–4.45

Total	4,921.77	571	11.60	10.69–12.59

**Figure:**
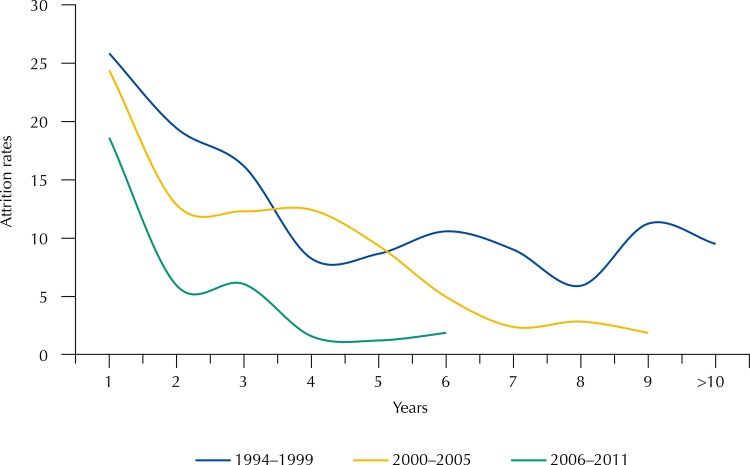
Attrition rates per 100 person-years estimated at different periods of the participants’ enrollment in a cohort of men who have sex with men, in 1994–1999, 2000–2005, 2006–2011, Project Horizonte.

An exploratory analysis of the data using statistical tests for medians (Mann-Whitney U test) and proportions (Chi-square test or Fisher’s exact test) was conducted by comparing the volunteers lost to follow-up with volunteers who remained in the study. Medians were shown with 25th and 75th percentiles (P25; P75). All independent variables presenting p < 0.25 in the univariate analysis were selected to construct the multivariate models.

The Kaplan-Meier curves followed by the Log-rank test were performed to compare each categorical variable with the outcome. The multivariate Cox proportional hazards model was used to adjust the factors associated with loss to follow-up. The strength of the association was assessed using the hazard ratio (HR; 95%CI). A step-by-step backward procedure was used to select the variables for the final model. Only adjusted variables showing a significant association (p < 0.05) with loss to follow-up remained in the final model. Schoenfeld residuals were used to assess the proportional hazards of the final model^12^. Statistical analyses of the data were performed using the software Stata, version 11.0 (Stata Corp., College Station, TX, USA).

The procedures of the Project Horizonte abide by Brazilian and International Research Ethics guidelines, and this study has been approved by the Research Ethics Committee of the Universidade Federal de Minas Gerais (Process 17750313.0.0000.5149). All subjects signed a free and informed consent form.

## RESULTS

A total of 1,197 MSM were enrolled in the Project Horizonte between 1994 and 2011. The median age of the volunteers when they enrolled in the cohort was 26 years (P25: 18 years; P75: 58 years). Among the volunteers, 23.1% had elementary school, 49.8% high school, and 27.8% college education. Considering monthly *per capita* income (Brazilian minimum wage), 10.9% received less than one Brazilian minimum wage, 49.2% received one to three Brazilian minimum wages, 22.3% received four to six Brazilian minimum wages, and 16.6% received more than six Brazilian minimum wages. Most participants had male partners (95.8%) in the last six months. Among those, only 28.8% had only steady partners, 33.1% only casual partners, and 33.9% both types of partnerships. Only 8.6% of the participants had sex with women. Approximately 24% reported practicing receptive anal intercourse without condoms with steady partners and 7.7% reported this practice with occasional partners without condoms. Among the volunteers, 10% reported bisexual practices (data not shown).

The median follow-up time for the volunteers was 2.5 years. During this period, 571 (47.7%) volunteers dropped out the study at different times. The median follow-up time of the subjects who dropped out the study was 1.46 years (P25: 0.25 years; P75: 3.38 years, ranging from 0.25 to 15.74 years). Among the individuals who remained in the study (626 subjects), the median follow-up time was 4.2 years (P25: 1.78 years; P75: 8.55 years, ranging from 0.25 to 17.3 years).

A total of 4,921.77 person-years were monitored from 1994 to 2011. The overall rate of loss to follow-up was 11.6/100 person-years. The highest rates were concentrated in the first three years of the study, with higher rate in the first year (22.91/100 person-years). After this period, the annual attrition rate remained below 9/100 person-years (Table 1).

Attrition rates were estimated at different periods of enrollment of the participants (Figure). Attrition rates presented a slight decline over the analyzed period. The number of person-years and attrition rates per period were: (a) 2,627.02 and 12.6/100 person-years (1994–1999), (b) 1,206.70 and 11.8/100 person-years (2000–2005), and (c) 1,088.07 and 9/100 person-years (2006–2011). It is noteworthy that, in the three periods evaluated, there were higher percentages of losses to follow-up in the first two years that tended to stabilize after the fourth year. The attrition rates decreased in the first year of each period: 25.84 (1994–1999), 24.39 (2000–2005), and 18.62 (2006–2011) per 100 person-years. The lowest percentages of loss to follow-up were observed in the third period analyzed (2006–2011).

Table 2 shows the sociodemographic and contextual variables of the participants who were in the follow-up and those who dropped out the study. The categorization into age groups revealed significant differences: a high percentage of loss to follow-up was observed in the age group of 21–30 years old (60.6%). Significant differences were observed in the following categories: *per capita* income, family income, places to find sexual partners, marital status, drug use, and willingness to participate in HIV vaccine trials. The proportion of loss to follow-up was higher among individuals with *per capita* and family incomes higher than six Brazilian minimum wages when compared to other income groups. The percentage of loss to follow-up was higher among individuals who went to places such as saunas, bars, nightclubs, and cruising areas to find sexual partners (70.5%). The proportion of loss to follow-up was higher among illicit drug users (21.1%). The loss to follow-up was higher for those who would not participate in HIV vaccine trials.

**Table 2:** Sociodemographic and contextual variables associated with loss to follow-up in a cohort of men who have sex with men. Project Horizonte, 1994–2011.

Variable	n	Remained in study n (%)	Loss to follow-up n (%)	p
Age group (years)	1,195			0.003
Up to 20		122 (19.6)	90 (15.8)	
21 to 30		316 (50.6)	346 (60.6)	
> 30		186 (29.6)	135 (23.6)	
Educational level	1,191			0.320
Elementary education		146 (23.6)	129 (22.1)	
High school		320 (57.6)	273 (47.8)	
College education		154 (24.8)	177 (31.0)	
Monthly *per capita* income of Brazilian minimum wage	1,184			0.000
1 to 3		358 (57.5)	230 (40.9)	
4 to 6		139 (22.3)	128 (22.7)	
> 6		67 (10.8)	132 (23.5)	
< 1		58 (9.3)	72 (12.8)	
Monthly family income of Brazilian minimum wage	1,192			0.000
1 to 3		198 (31.9)	93 (16.3)	
4 to 6		89 (14.3)	92 (16.1)	
> 6		74 (11.9)	167 (29.2)	
< 1		52 (8.4)	49 (8.6)	
Unknown		208 (33.5)	170 (29.8)	
Number of dependents	1,192			0.220
Up to 2		355 (56.9)	303 (53.4)	
> 2		269 (43.1)	264 (46.6)	
Marital status	1,189			0.010
Single		579 (93.1)	544 (95.9)	
Other		43 (6.9)	23 (4.1)	
Places to look for sexual partners (sauna, bar)	1,188			0.030
No		221 (35.4)	166 (29.5)	
Yes		404 (64.6)	397 (70.5)	
Received money in exchange for sexual relation	1,193			0.29
No		601 (96.3)	541 (95.1)	
Yes		23 (3.7)	28 (4.9)	
Alcohol intake during flirting or sex	1,194			0.880
Yes		462 (74.0)	420 (73.3)	
No		162 (26.0)	150 (26.3)	
Drug use	1,191			0.000
Yes		83 (13.3)	120 (21.1)	
No		539 (86.7)	449 (78.9)	
Willingness to participate in future HIV vaccine trials	1,195			0.006
Yes		373 (59.7)	294 (51.6)	
No		94 (15.0)	121 (21.2)	
Depends/Does not know		158 (25.3)	155 (27.2)	

Table 3 shows the characteristics of sexual behavior and sexual partners in the past six months. Higher proportion of loss was found among volunteers that reported bisexual practices. Regarding consistent condom during receptive anal sex, higher proportion of loss to follow-up was found among those who did not use condoms regularly (26.8%).

**Table 3:** Characteristics of sexual behavior associated with loss to follow-up in a cohort of men who have sex with men. Project Horizonte, 1994–2011.

Variable	n	Remained in study n (%)	Loss to follow-up n (%)	p
Male partners*	1,193			0.170
No		24 (3.9)	27 (4.7)	
Steady and casual		199 (32.0)	205 (35.9)	
Only steady		195 (31.3)	148 (25.9)	
Only casual		204 (32.8)	191 (33.4)	
Female partners*	1,196			0.000
No		595 (94.7)	501 (87.7)	
Steady and casual		8 (1.3)	15 (2.6)	
Only steady		10 (1.6)	17 (3.0)	
Only casual		15 (2.4)	38 (6.7)	
Number of steady partners*	1,196			0.800
Not applicable		228 (36.5)	218 (38.2)	
1		347 (55.5)	306 (53.6)	
> 1		50 (8.0)	47 (8.2)	
Number of casual partners*	1,189			0.220
Not applicable		220 (35.3)	175 (31.0)	
1 to 2		118 (18.9)	118 (20.0)	
3 to 6		135 (21.6)	144 (25.4)	
> 6		151 (24.2)	128 (22.6)	
Sexual behavior*	1,193			0.005
Homosexual		570 (91.5)	475 (83.4)	
Bisexual		35 (5.6)	84 (14.7)	
Absence of sex		18 (2.9)	11 (1.9)	
Inconsistent use of condoms during receptive anal sex*	1,193			0.001
No		206 (33.0)	253 (44.5)	
Yes, with a steady partner		166 (26.6)	120 (21.1)	
Yes, with a causal partner		57 (9.1)	35 (6.2)	
Yes, with steady and casual partner		20 (3.2)	14 (2.5)	
Absence of anal intercourse		176 (28.1)	146 (25.7)	
Inconsistent use of condoms during sex*	1,192			0.100
No		237 (37.9)	257 (45.6)	
Yes, with a steady partner		167 (26.7)	142 (25.0)	
Yes, with a causal partner		50 (8.0)	33 (5.8)	
Yes, with steady and casual partner		17 (2.7)	14 (2.5)	
Absence of anal intercourse		154 (24.6)	121 (21.3)	
Consistent use of condoms during receptive anal sex*	1,195			0.008
Yes		324 (51.8)	288 (50.5)	
No		125 (20.0)	153 (26.8)	
Absence of receptive anal sex		176 (28.2)	129 (22.6)	
Consistent use of condoms during insertive anal sex*	1,193			0.150
Yes		658 (55.2)	311 (54.7)	
No		263 (22.0)	138 (24.2)	
Absence of receptive anal sex		272 (22.8)	120 (21.1)	

* Data refer to the six months before the interview.

All the variables presenting p < 0.25 in the univariate analysis (Tables 2 and 3) were selected to construct the multivariate Cox regression model (Table 4). The risk factors associated with losses to follow-up were: age group of 21–30 years old (HR = 1.43; 95%CI 1.13–1.81), monthly *per capita* income higher than six or below one Brazilian minimum wage (HR = 1.74; 95%CI 1.13–1.81), having more than two dependents (HR = 1.26; 95%CI 1.06–1.50), bisexual practices (HR = 2.05; 95%CI 1.60–2.63), and inconsistent condom use during receptive anal sex (HR = 1.50; 95%CI 1.23–1.83).

**Table 4:** Cox model for losses to follow-up in a cohort of men who have sex with men. Project Horizonte, 1994–2011.

Variables	Univariate		Multivariate	
	
	HR (95%CI)	p	HR (95%CI)	p
Age group (years)				
Up to 20	1		1	
21 to 30	1.33 (1.05–1.67)	0.02	1.43 (1.13–1.81)	0.003
> 30	0.94 (0.72–1.23)	0.66	1.03 (0.78–1.36)	0.81
Monthly *per capita* income				
1 to 6	1		1	
> 6 or < 1	1.72 (1.45–2.05)	0.000	1.74 (1.13–1.81)	0.000
Number of dependents				
Up to 2	1		1	
> 2	1.26 (1.07–1.49)	0.006	1.26 (1.06–1.50)	0.007
Have children				
No	1		1	
Yes	3.00 (1.72–5.23)	0.000	2.03 (1.13–3.63)	0.020
Sexual behavior				
Homosexual	1		1	
Bisexual	2.30 (1.82–2.90)	0.000	2.05 (1.60–2.63)	0.000
Not applicable	0.67 (0.37–1.21)	0.180	0.75 (0.40–1.39)	0.365
Use of condoms during receptive anal sex*				
Yes	1		1	
No	1.23 (1.00–1.50)	0.050	1.50 (1.23–1.83)	0.000
Use of condoms during insertive anal sex*	1			
Yes	1.44 (1.18–1.75)	0.000		
No	0.92 (0.75–1.13)	0.420		
Drug use*	1			
No	1.44 (1.17–1.76)	0.000		
Yes				
Willingness to participate in future HIV vaccine trials				
Yes	1.27 (1.03–1.57)	0.020		
No	1.21 (0.99–1.47)	0.050		
Depends/Does not know	1			

* Data refer to the six months before the interview.

## DISCUSSION

The findings of this study contribute to a better understanding of the attrition rates and factors associated with the loss to follow-up among MSM, participants of the long term cohort study. The overall attrition rate observed in the Project Horizonte in the study period (1994–2011) was 11.6/100 person-years. The analysis of different enrollment periods showed slight decreases in the loss to follow-up over time, with attrition rates of 12.6/100 person-years (1994–1999), 11.8/100 person-years (2000–2005), and 9/100 person-years (2006–2011). The main factors associated with loss to follow-up are linked to the age group of 21–30 years old, monthly income of more than six or less than one Brazilian minimum wage, having more than two dependents, report of bisexual practice, and inconsistent use of condoms for receptive anal sex.

Few studies have assessed attrition and retention rates in the MSM population. The studies generally report the proportion of retention. In the Multicenter AIDS Cohort Study (MACS), the retention rate was 88.5% in 9.5 years of follow-up^3^. The Brazilian Praça Onze cohort study monitored seronegative MSM and reported retention rates of 97%, 91%, and 88% at six, 12, and 18 months of follow-up^13^, respectively. An American study with Latino population of homosexuals/bisexuals and transgender persons reported retention rates of 83% and 80% in three and six months of follow-up, respectively^6^. A cohort study conducted in China showed that 86.2% of the participants completed the 12 months of follow-up^14^. Because of methodological differences, it is difficult to compare the results of the Project Horizonte with the aforementioned cohorts. The Project Horizonte is an open cohort with longer duration, unlike the studies mentioned, which are closed cohorts with shorter follow-up periods. Additionally, the rate of loss to follow-up calculated in Project Horizonte considered the time that each participant contributed to the study, i.e., the time from recruitment to loss to follow-up.

In this study, the highest attrition rate was observed in the first years of follow-up, and retention was higher after this period. This result is similar to other cohort studies with MSM^3^ ^,^ ^13^ ^,^ ^15^, which indicates that retention rate is higher after the initial period of the study and remains stable over time. It is possible that the connection and commitment to the study were not well established in the first years of participation.

Age is an important demographic determinant associated with loss to follow-up usually described in the literature. Longitudinal studies conducted among MSM reported that the population under the age of 30 years was associated with losses to follow-up^3^ ^,^ ^16^. Likewise, this study showed a greater loss of volunteers aged 21–30 years. Similar results were found in a previous analysis conducted with volunteers of the Project Horizonte ^2^. One explanation is that young participants might be committed to their studies or might work night shifts that coincide with the Project Horizonte office hours. Thus, the lack of available time may contribute to the loss to follow-up. We must also consider that the perception of risks among these young persons is built on a historical moment when AIDS is no longer seen as a threat. Furthermore, the sense of invulnerability that is common in this age group leads them to shift the responsibility for a possible infection to others and not to themselves. For several young persons, the systematic participation in health research, knowledge about prevention, and the decision about condom use presuppose a complex reflection on their responsibility for the risk, which is often not yet established.

In the Project Horizonte, retention was lower among volunteers with extreme upper and lower income ranges (less than one and more than six Brazilian minimum wages) and with more than two dependents. Retention rates were associated with income in a study conducted with MSM enrolled in a counseling program; the authors observed that low-income individuals are less likely to complete the study^8^. Associations between loss to follow-up and high incomes were not observed in other studies conducted with MSM.

Inconsistent use of condoms during receptive anal sex was also associated with loss to follow-up. In a study conducted with MSM in the United States, individuals who prematurely dropped out the study reported having unprotected anal sex compared with those who either dropped out the study later or completed the follow-up^8^. Different results were found in an online prospective study with MSM^15^, which identified unprotected anal sex as predictors of retention. It is possible that the volunteers who remained in the Project Horizonte, are more likely to participate in health research and more easily incorporate safe practices in their sexual lives. In addition, volunteers who practice risky activities might be aware that unprotected sexual practices are incompatible with the preventive profile of the Project Horizonte and, therefore, drop out the study because of difficulty or embarrassment in reporting the recurrent risks.

Volunteers who reported bisexual practices were twice as more likely to drop out the study than those who reported exclusively homosexual practices. The predictors of nonparticipation in a prevention program for MSM in the United States showed that bisexuals were almost twice as more likely to be among the non-responders than those who identified themselves as homosexual^17^. A study addressing bisexual behavior and factors associated with HIV among young Chinese MSM found that persons who reported bisexual behavior were less likely to participate in HIV prevention activities^18^. This group of persons probably still uses strategies to hide their behavior to prevent discrimination and verbal and physical abuse and, therefore, maintain their appearance in accordance with the social concept of hegemonic masculinity^19^. These results corroborate a study that reported that bisexual men felt obliged to maintain a private sexual identity because they see bisexuality as a stigmatized identity^20^. In addition, Miskolci^21^ has stated there has been a significant transformation in the way that persons of the same sex express their desires in contemporary society. Homosexual relationships are no longer prohibited as long as they do not threaten heterosexual hegemony^21^. From this presumption, there may be a stronger link between low visibility and discretion of bisexual individuals and moral restriction put upon them by heterosexual rules in the name of sociability.

The results of this study exposed some limitations that could affect the Project Horizonte. Differences were observed among volunteers who dropped out and those who remained in the cohort study. The individuals who dropped out were more vulnerable in comparison with those who remained in the study, suggesting differential bias. The volunteers more likely to drop out the study reported inconsistent use of condoms during receptive anal sex, which may underestimate the incidence rates of HIV infection and other sexual transmitted infections in the cohort population. Two groups showed significant rates associated with loss to follow-up: young persons aged 21–30 years and those who reported bisexual practices. Considering the current high incidence of infection among young homosexuals and the difficulty to reach the bisexual population in health research, it is likely that these two groups are not being fully evaluated in the Project Horizonte. Limitations of this analysis are related to a possible violation of the assumptions of survival analysis; analysis to assess the proportional hazards of the final model was performed and attrition rates were estimated considering different admission periods.

The highest rates of loss to follow-up occurred in the first three years of the study. It should be noted that the characteristics of those who came only to the first visits of the Project showed that a large proportion of them more than likely never had any intention of participating in the cohort. Nonetheless, this group most probably represents the population that would volunteer in cohort studies with MSM.

Similar to other studies^4^ ^,^ ^14^ ^,^ ^22^ ^-^ ^25^, the Project Horizonte has several strategies to maintain the monitoring of the volunteers, such as: helping with transportation and food costs, sending letters, making phone calls and sending e-mails for scheduling appointments, and sending event invitations and holiday cards. Currently, the access to volunteers and the search process is being facilitated by the use of social networks because of their speed and continuous flow. In our view, in recent years new technologies are allowing more space and visibility for the gay community, so it is necessary to invest and expand the use of theses universes (e.g., social networks, mobile applications) as a strategy for recruiting and retaining volunteers, incorporating more specific language to this group.

Finally, the participation in educational activities (i.e., discussion forums and seminars) and the development of a volunteer team for dissemination and recruitment expand the role of the study participants. Currently, the Project Horizonte makes efforts to maintain the periodic visits of volunteers to ensure retention rather than evaluate the real reasons for the losses to follow-up. The rescue of those lost to follow-up is difficult and requires strategies that target the characteristics of the MSM population.

In general, studies on loss to follow-up are limited to the analysis of factors that allow comparability of data, particularly sociodemographic variables. This study showed that, in addition to sociodemographic variables, other variables are important for assessment of losses, especially those in the sociocultural context. The determinants of losses to follow-up have multiple aspects, and their real reasons are revealed in subjective questions to the participants, suggesting qualitative analysis to better understand this issue. For greater effectiveness, retention strategies must be reassessed considering the relationship between the particular characteristics of the MSM population and the motivations to engage with health research.
